# Can SARS‐CoV‐2 infection trigger rheumatoid arthritis? A case report

**DOI:** 10.1002/ccr3.5748

**Published:** 2022-04-18

**Authors:** Sirine Bouzid, Kawther Ben Abdelghani, Saoussen Miledi, Alia Fazaa, Ahmed Laatar

**Affiliations:** ^1^ Department of Rheumatology Mongi Slim Hospital Tunis Tunisia; ^2^ Faculty of Medicine of Tunis Tunis El Manar University Tunis Tunisia

**Keywords:** autoimmune diseases, case report, COVID‐19, rheumatoid arthritis

## Abstract

Inflammatory arthritis has been reported after SARS‐COV‐2 infection. We present a case of a 38‐year‐old female patient who developed polyarthralgia 1 month after SARS‐COV‐2 infection. Musculoskeletal examination was significant for synovitis of hands and wrists. Antinuclear antibody (ANA), rheumatoid factor (RF), and anti‐cyclic citrullinated peptide (CCP) antibodies were positive. Magnetic resonance imaging of the hands showed synovitis of the metacarpophalangeal joints and proximal interphalangeal joints of the hands, wrist joints, and tendinitis with tenosynovitis. The patient was diagnosed with seropositive nonerosive rheumatoid arthritis (RA) and initiated on therapy using nonsteroidal anti‐inflammatory agents and disease‐modifying anti‐rheumatic drug methotrexate leading to an improvement in symptoms.

## Abstract 2

Cases of autoimmune and auto‐inflammatory diseases triggered after COVID‐19 have been reported. We report the case of a 38‐year‐old female patient who developed seropositive, nonerosive rheumatoid arthritis (RA) one month after SARS‐CoV‐2 infection.

## 1 INTRODUCTION

Coronavirus disease 19 (COVID‐19) is a viral infection caused by severe acute respiratory syndrome coronavirus 2 (SARS‐COV‐2). An increase in pro‐inflammatory cytokines (IFN‐γ, IL‐1, IL‐6, IL‐12, and TNF‐α) and chemokines (CCL2, CXCL10, CXCL9, and IL‐8) described as a cytokine storm or hyperinflammatory syndrome may develop during the course COVID‐19.^1^ These pro‐inflammatory cytokines are targets in many of our inflammatory arthritis including RA.

Cases of autoimmune disorders presenting or exacerbating after SARS‐Cov‐2 infection have been reported.^2^ Herein, we report a case of RA occurring one month after COVID‐19 infection.

## 2 PATIENT INFORMATION

The patient is a 38‐year‐old female with no known medical conditions.

### 2.1 Clinical findings

She presented to the rheumatology clinic for the first time with the chief complaint of 4 months of progressively worsening symmetric pain in the small joints of her hands, wrists, knees, and ankles. She reported early morning stiffness lasting for 2 h every day with complaints of severe fatigue all day long. She was diagnosed with a COVID‐19 infection a month prior, as confirmed by the SARS‐COV‐2 PCR performed on her nasal swab. Her COVID‐19 symptoms were very mild and manifested as fever with chills, cough not needing oxygen support or hospitalization, and mechanical ventilation. At the time, she was not yet vaccinated against COVID‐19. On the day of her visit to the rheumatology clinic, she did not report fever, chills, night sweats, weight loss, rash, and loss of appetite. There were no preceding genitourinary infections including chlamydia or gonorrhea, and no preceding gastrointestinal infections either. The patient has no history of psoriasis and/or uveitis. The patient does not smoke, use nicotine in any form or drink alcohol, or use recreational drugs. She had a maternal aunt who had RA.

### 2.2 Diagnostic assessment

The inflammatory markers included a normal erythrocyte sedimentation rate of 13 mm/hour and C‐ reactive protein of 4.3 mg/liter. Immunological workup revealed elevated antinuclear antibodies titer (1:640; normal range 1:80), negative extractable nuclear antigen, and double‐stranded DNA antibodies. The patient, however, had a positive rheumatoid factor measured by ELISA (62.29 IU/ml; normal range <20 IU/ml) and a positive anti‐cyclic citrullinated peptide (anti‐CCP) antibody (237.39; normal range <20 units). Serologies for hepatitis B virus and hepatitis C virus were negative. Radiologic images including X‐rays of the hands, knees, ankles, and feet were all negative. We performed an ultrasound of the hands, and there was no evidence of joint damage and destruction. However, magnetic resonance imaging showed synovitis of the bilateral distal radio‐ulnar joints and second metacarpophalangeal joint of the left hand. It also showed inflammation around the extensor carpi ulnaris tendon with the synovial enhancement of the tendon sheath suggestive of tenosynovitis. No adjacent osseous destruction was found.

### 2.3 Diagnosis

Although our patient presented with inflammatory arthritis 1 month after a viral infection (COVID‐19), it is less likely to be reactive arthritis since she presented with symmetric polyarthritis of small and large joints without increased inflammatory markers (ESR and CRP) and had positive rheumatoid factor and anti‐CCP antibodies. Our patient meets the 2010 ACR/EULAR formal criteria for the classification of RA, which includes joint involvement of more than 10 joints for more than 6 weeks with high positive anti‐CCP antibodies. No striking extra‐articular signs or symptoms were found to suggest different systemic immune diseases such as systemic lupus erythematosus (SLE). Disease activity was moderate with a DAS28‐ESR of 4.6.

### 2.4 Therapeutic interventions

Treatment was initiated with methotrexate and nonsteroidal anti‐inflammatory drugs.

### 2.5 Follow‐up and outcome of interventions

After treatment, there was patient‐reported improvement in symptoms and objective improvement in disease activity with DAS‐28 ESR of 3.7 at 2 months and 3.1 at 5 months.

## 3 DISCUSSION

Rheumatoid arthritis is an immune‐mediated disease, where genetic predisposition, environmental factors including smoking, infections, and hormonal factors all play a major role. The relationship between RA and infections is due to the potential role of microbes in producing arthritis either by the colonization of the joints by the pathogen or by an aberrant autoimmune reaction produced by the host response to the infection.^3^ Some studies have linked respiratory viral infections, particularly parainfluenza and coronavirus to be associated with a number of incident RA.^4^ In a large Korean study, the authors noted that infections with viruses including endemic human coronavirus and parainfluenza virus coincided with an increase in the rate of development of RA. The hypothesis is that initial respiratory viral infections involve the oral mucosa and lungs; this is relevant to the generation of immune response and future RA.^5^ However, no such cases or reports to indicate that patients who are infected by SARS‐COV‐2 or any other human coronavirus develop RA.^4^ Since the emergence of COVID‐19, cases of reactive arthritis post‐SARS‐CoV‐2 infection have been reported.^6^, ^7^ This low frequency can be explained by the use of corticosteroids in the treatment of this viral infection.^8^ Of all the cases reported so far, the majority are male, joint inflammation has predominantly affected lower extremities, and symptoms have started 12–32 days after the COVID‐19 infection.^8^ Hence, reactive arthritis remains high on the differential in these cases. Previous cases of ACPA‐positive arthritis following COVID‐19 were also reported.^9^, ^10^, ^11^ In a study by Derksen et al., authors tried to determine the seroprevalence of ACPA after COVID‐19.^12^ ACPA was measured in 61 patients 5 weeks after hospitalization; only two patients tested positive for ACPA. These two patients were already previously diagnosed with ACPA‐positive RA. Thus, ACPA positivity was not increased after COVID‐19.^12^

The development of systemic lupus erythematosus (SLE), following the infection with SARS‐CoV‐2, has been described.^2^ Most cases reported were women with a median age of 43 years (range 18–85).^2^ SLE was suspected when there were symptoms of autoimmune thrombocytopenia, serositis, and renal disease, and there was a paucity of respiratory disease. Glucocorticoids alone or in association with hydroxychloroquine were used as the first‐line treatment.^2^ Autoantibody production is implicated in the pathogenesis of SLE, and these autoantibodies are triggered due to the COVID‐19 infection.^13^ In a meta‐analysis, patients (*n* = 120) hospitalized for COVID‐19 underwent immunological workup, and the prevalence of ANA and RF were 32.1% and 19.9%, respectively.^14^ Other autoantibodies have also been reported but at lower frequencies; for example, the prevalence of anti‐CCP antibodies is only 5.46%.^14^, ^15^, ^16^

This case highlights the ability of SARS‐CoV‐2 to trigger autoimmune phenomena, and the potential mechanisms include molecular mimicry, epitope spreading, bystander activation, release of encrypted host antigens from tissues, and activation of superantigens.^17^

## 4 CONCLUSION

We report a case of RA starting one month after a COVID‐19 infection. Further case reports and case series are needed to comprehend the link between SARS‐Cov‐2 and autoimmune diseases, particularly RA.

## CONFLICTS OF INTEREST

None.

## AUTHOR CONTRIBUTION

Sirine Bouzid has drafted the work. Kawther Ben Abdelghani has substantively revised the work. Saousen Miledi and Alia Fazaa have made substantial contributions to the literature research. Ahmed Laatar has made substantial contributions to the conception of the work.

## ETHICAL APPROVAL

None.

## CONSENT

The patient gave her consent for publishing the case with absolute respect of anonymity.
